# Global Identification of *ANTH* Genes Involved in Rice Pollen Germination and Functional Characterization of a Key Member, OsANTH3

**DOI:** 10.3389/fpls.2021.609473

**Published:** 2021-04-13

**Authors:** Su Kyoung Lee, Woo-Jong Hong, Jeniffer Silva, Eui-Jung Kim, Soon Ki Park, Ki-Hong Jung, Yu-Jin Kim

**Affiliations:** ^1^Graduate School of Biotechnology, Crop Biotech Institute, Kyung Hee University, Yongin, South Korea; ^2^School of Applied Biosciences, Kyungpook National University, Daegu, South Korea; ^3^Department of Life Science and Environmental Biochemistry, Life and Industry Convergence Research Institute, Pusan National University, Miryang, South Korea

**Keywords:** AP180 N-terminal homology protein, endocytosis, gene editing (CRISPR-Cas9), *Oryza sativa*, pollen germination

## Abstract

Pollen in angiosperms plays a critical role in double fertilization by germinating and elongating pollen tubes rapidly in one direction to deliver sperm. In this process, the secretory vesicles deliver cell wall and plasma membrane materials, and excessive materials are sequestered via endocytosis. However, endocytosis in plants is poorly understood. AP180 N-terminal homology (ANTH) domain-containing proteins function as adaptive regulators for clathrin-mediated endocytosis in eukaryotic systems. Here, we identified 17 ANTH domain-containing proteins from rice based on a genome-wide investigation. Motif and phylogenomic analyses revealed seven asparagine-proline-phenylalanine (NPF)-rich and 10 NPF-less subgroups of these proteins, as well as various clathrin-mediated endocytosis-related motifs in their C-terminals. To investigate their roles in pollen germination, we performed meta-expression analysis of all genes encoding ANTH domain-containing proteins in *Oryza sativa* (*OsANTH* genes) in anatomical samples, including pollen, and identified five mature pollen-preferred *OsANTH* genes. The subcellular localization of four OsANTH proteins that were preferentially expressed in mature pollen can be consistent with their role in endocytosis in the plasma membrane. Of them, OsANTH3 represented the highest expression in mature pollen. Functional characterization of *OsANTH3* using T-DNA insertional knockout and gene-edited mutants revealed that a mutation in *OsANTH3* decreased seed fertility by reducing the pollen germination percentage in rice. Thus, our study suggests OsANTH3-mediated endocytosis is important for rice pollen germination.

## Introduction

Like all eukaryotes, plant cells are composed of intracellular compartments with proteins located either inside or in the plasma membrane (PM). Thus, the precise transport of proteins and localization of certain proteins are critical for the normal function of cells, requiring complex and systematic regulation of membrane trafficking (Zouhar and Sauer, 2014). The balanced processes of endocytosis and exocytosis/secretion maintain proteins and lipids for the PM in addition to regulating transport and signaling. Endocytosis is defined as the uptake of membrane-associated and soluble cargo from the extracellular space through the formation of a vesicle at the PM. Due to its applications in human health, endocytosis has been extensively studied in yeast and mammalian cell systems, compared with limited recent investigations in plants (Robinson et al., 2008; Reynolds et al., 2018).

When pollen grains land on the stigma in higher plants, compatible grains germinate, and an elongating pollen tube forms and grows into the style toward the ovules. Germination of pollen grains is a complex biological event that requires numerous factors and activities (Wang et al., 2010). Many of these factors, such as nutrients, proteins, signaling molecules, and various cell components, are contained in vesicles. The vesicles carrying cargo molecules are involved in several stages of endocytic pathways, of which clathrin-mediated endocytosis (CME) is one of the major endocytic activities (Lacy et al., 2018). The process of pollen germination is essential for successful reproduction in plants; however, little is known regarding the mechanism of selective internalization or molecular sorting during pollen germination.

Endocytosis processes include various steps, such as cargo collection, vesicle assembly and invagination from the PM, and trafficking to the trans-Golgi network (TGN)/early endosome (EE), that are mediated by coat proteins as well as adaptor and accessory proteins (Nguyen et al., 2018). Clathrin, which functions as a coat protein during vesicle formation, does not directly interact with cargoes; instead, it interacts via adaptor proteins. The epsin N-terminal homology (ENTH) domain acts as an adaptor during CME in animal cells. Proteins with a similar domain, the AP180 N-terminal homology (ANTH) domain, also function in clathrin-coated vesicle (CCV) formation and cargo sorting at the PM during CME (Duncan and Payne, 2003; Legendre-Guillemin et al., 2004). In ANTH domain-containing proteins (from here on referred to as ANTH proteins encoded by *ANTH* genes), the N-terminus of the ANTH domain is located in the PM and possess affinity to phosphatidylinositol 4,5-biphosphate, and the C-terminus is involved in CCV formation in combination with other elements such as clathrin (Itoh et al., 2001; Zouhar and Sauer, 2014). The yeast AP180 protein functions at the budding region of the PM (Legendre-Guillemin et al., 2004), and in neurons the AP180 proteins are enriched in the presynaptic terminal membrane (Yao et al., 2002), suggesting their association with polar growth.

Plants contain a larger number of *ANTH* genes than mammals or yeast; however, only a few have been shown to function in cargo trafficking (Barth and Holstein, 2004; Holstein and Oliviusson, 2005; Zouhar and Sauer, 2014). AtAP180/PICALM6 has been extensively shown to interact with clathrin and accelerate cargo efficiency *in vitro* (Barth and Holstein, 2004). *Arabidopsis* epsin-like clathrin adaptor (AtECA) protein 1, AtECA2/PICALM5a, and PICALM4 have shown involvement in cytokinesis during cell plate expansion (Song et al., 2012; Nguyen et al., 2018). Recent studies demonstrated the role of ANTH domain-containing proteins in *Arabidopsis* pollen tube growth, such as acting as adaptors of CCV or engaging in pollen tube PM integrity (Muro et al., 2018; Kaneda et al., 2019). PICALM5b and AtECA2/PICALM5a are involved in maintaining pollen tube integrity and tip localization of receptor kinases during CME (Muro et al., 2018). Endocytosis adaptor of pollen tube 1 (EAP1) interacts with REN4 (WD40 domain protein) and REN4 interacting partner 1 (ROP1) and then removes both proteins from the PM by CME to maintain polar tip growth of the pollen tube (Li et al., 2018).

The role or even the existence of the *ANTH* gene family in monocot plants is not well understood. In this study, we investigated the genome-wide characteristics of ANTH domain-containing proteins in *Oryza sativa* (rice), named OsANTH, and performed domain and phylogenetic analyses with *Arabidopsis* ANTH proteins. The highest expressed gene in rice pollen, *OsANTH3*, was further analyzed through characterization of the T-DNA insertional knockout mutant and gene-edited mutants. These findings are expected to contribute to a comprehensive understanding of the ANTH family in rice and their role in late pollen development.

## Materials and Methods

### Plant Materials and Growth Conditions

Two japonica rice (*Oryza sativa subsp.* japonica) varieties, Dongjin (DJ) and Nipponbare (NP), as the wild-type (WT) plants, together with the T-DNA insertional mutants in the DJ background were grown in either a growth chamber under a day/night cycle of 28°C (16 h light) and 22°C (8 h dark) with ∼80% humidity or a living modified organism-controlled paddy field (Kyung Hee University, Suwon, South Korea) for the phenotype analysis. Anthers from the WT (DJ) plants were harvested at different developmental stages for quantitative real-time polymerase chain reaction (qRT-PCR) analysis as previously described (Moon et al., 2019).

### Multiple Sequence Alignment and Phylogenetic Tree Construction

To perform phylogenetic analysis of the ANTH proteins in rice and *Arabidopsis*, we collected protein sequences with locus ID from the Rice Genome Annotation Project^1^, National Center for Biotechnology Information (NCBI^2^), and Phytozome platform^3^. Multiple alignment of the amino acid sequences was performed using ClustalW. The phylogenetic analysis was performed using MEGA 7.0.26 with maximum likelihood and neighbor-joining methods (bootstrap repeat was 1,000) as previously described (Kim et al., 2019).

### Meta-Expression Analysis

We used a publicly available rice Affymetrix microarray data set prepared from anthers and pollen from the NCBI Gene Expression Omnibus (GEO) (Hong et al., 2019) to identify late pollen-preferred genes. To examine expression patterns, we used the Affy package encoded by R language to normalize the signal intensity and then transformed them into log2 values. The normalized data, including averaged Affymetrix anatomical meta-expression data, were then used for further investigation, i.e., heatmap construction and identification of the late pollen-preferred genes (Nguyen et al., 2015).

Microarray data, including Affymetric and Agilent array data, were downloaded from the NCBI GEO^4^ and Genevestigator^5^ (Zimmermann et al., 2004; Cao et al., 2012). We then uploaded the normalized data to the Multi Experiment Viewer^6^ and visualized the data using heatmaps. We used Genevestigator to compare the levels of gene expression in several organs and to estimate the functional similarity among rice and *Arabidopsis* members.

### RNA Extraction, cDNA Construction, and qRT-PCR

Tissue samples, including pollen of rice (*Oryza sativa* cv. Dongjin) grown in paddy fields, were frozen in liquid nitrogen and homogenized with a TissueLyser II (Qiagen, Hilden, Germany). Total RNA was extracted using RNAiso Plus according to the manufacturer’s protocol (TakaraBio, Kyoto, Japan). cDNAs were synthesized using a SuPrimeScript RT premix from GeNet Bio (Chungcheongnam-do, South Korea) (Vo et al., 2015). qRT-PCR was performed with a Qiagen Rotor-Gene Q qRT-PCR cycler (Qiagen) using the following thermal cycling procedure: 95°C for 10 s, 60°C for 30 s, and 72°C for 1 min. To evaluate tissue-specific expression patterns by qRT-PCR, we used rice ubiquitin 5 (*OsUbi5*, *LOC_Os01g22490*) (Jain et al., 2006) as an endogenous control to normalize the variance in the amount of cDNA. Gene-specific qRT-PCR primers were designed for a specific region of each gene (Supplementary Figure 1 and Supplementary Table 2), and the accuracy and efficiency of each primer set were verified through PCR amplification of the gDNA to optimize the PCR conditions and melting curve. The fold change was calculated using the comparative delta-CT (Pfaffl, 2001; Silver et al., 2006). Three biological replicates were analyzed, and each reaction was performed at least three times with technical replicates.

### Subcellular Localization Analysis

The coding sequences (CDSs) of four *OsANTH* genes were amplified from mature anther cDNA and cloned into pGreen vectors fused with C-terminal green fluorescence protein (GFP). All the cloning primers used in the experiments are listed in Supplementary Table 2. The constructs were transfected into *Agrobacterium tumefaciens* strain GV3101 and used for *Nicotiana benthamiana* infiltration. Two to three days after infiltration, GFP fluorescence was observed with a confocal laser scanning microscope (Zeiss LSM 510; Zeiss, Oberkochen, Germany) using 488/505–530 nm excitation/emission filter sets. Fluorescence images were digitized using the Zeiss LSM image browser. To identify the subcellular localization in the cell membrane, the tobacco leaves were stained with FM4–64 (Thermo Fisher Scientific, Waltham, MA, United States). Tobacco leaf discs were immersed in 0.1% FM4–64 solution for more than 15 min under dark conditions and observed in a red fluorescence protein (RFP) channel using 558 nm excitation/emission filter sets. Brefeldin A (BFA) treatment was performed for approximately an hour after the tobacco leaf was immersed in 10 μM BFA.

### Isolation and Analysis of Mutant Plants

A T-DNA insertional mutant line of *OsANTH3* was isolated from the T-DNA insertional mutant population in the PFG library (Hong and Jung, 2018). Genotypes were determined via PCR in 50 μL of a mixture containing 20 ng of genomic DNA, 10X e-Taq buffer, 0.2 mM dNTP, 0.5 U of e-Taq polymerase (Solgent, Daejeon, Korea), and 1 mM of the primers. The protocol included 35 cycles at 94°C for 60 s, 60°C for 60 s, and 72°C for 120 s. The primers used for genotyping were 5′-CACATCTGGGTGGGAGCTTG-3′ (3,276 bp downstream from the ATG start codon of the *ANTH3* gene), 5′-GCCATTGGTACGAGGTCACC-3′ (4,101 bp downstream from the ATG start codon of *ANTH3* gene), and 5′-AACGCCTGATCAATTCCACAG-3′ (80 bp downstream from the ATG start codon of the *GUS* gene).

Additional mutant alleles were generated using the CRISPR/Cas9 system. The specific target sequences were identified using the online CRISPRdirect program (Naito et al., 2015). The oligomers were cloned into the *Bsa*I site of the pRGEB32 vector, which was then used to transform the WT rice via *Agrobacterium* (Kim E. et al., 2020). At least two regions were selected for gene editing, and approximately 20 hygromycin-selected lines were analyzed. Gene editing was confirmed by Sanger sequencing.

Histochemical GUS-staining was performed as described by Moon et al. (2019). The assayed flowers and pollen grains were photographed with an Olympus BX61 microscope (Olympus, Tokyo, Japan).

### Cytological Analysis of Pollen Germination

Flowers and anthers were photographed with a SZX61 microscope (Olympus). To investigate pollen maturation, spikelets were collected before anthesis, and the pollen grains were squeezed out with tweezers and stained with 1% I_2_-KI.

To observe the *in vitro* pollen germination percentage and morphology of pollen tubes, fresh pollen grains collected just after anthesis were placed directly onto a solid or liquid pollen germination medium (PGM). The PGM was freshly prepared with 20% (w/v) sucrose, 10% polyethylene glycol 4000, 3 mM calcium nitrate, 40 mg/L boric acid, and 10 mg/L vitamin B1 (pH 6.8–7.0) (Kim E. et al., 2020). The solidified PGM slide with 1% agarose was covered with a cover glass and incubated in a moist chamber at 28°C in the dark for 20 min. The germination percentage was analyzed in triplicate with at least 150 pollen grains in each examination.

Ikarugamycin (IKA) has been used as a CME inhibitor in plants and animals, although its mode of action remains to be determined (Elkin et al., 2016). For the IKA treatment, different concentrations (10, 50, and 100 μM) were prepared in PGM using a 10 mM IKA stock solution prepared in dimethyl sulfoxide (DMSO). In addition, pollen grains were germinated in the presence of DMSO as control. Working concentrations of DMSO were < 0.2% (v/v).

### Yeast Two Hybrid Analysis

The full length, N-terminal (1∼1,095 bp), and C-terminal (1,096∼end) CDSs of ANTH3 were cloned at the *BamH*I site of the pGADT7-Rec vector. The full-length, N-terminal, and C-terminal CDSs of *GORI* were cloned at the *EcoR*I/*BamH*I site of the pGBKT7 vector (Kim et al., 2021). The C-terminal (633-end) CDS of OsPME31 (Kim Y. et al., 2020) was cloned into the pGBKT7 vector. To test the self-transcriptional activity of the GORI bait, the plasmid was introduced with an empty prey vector into the yeast strain AH109. Yeast transformants of the bait and prey plasmids were selected on a synthetic defined (SD) minimal medium lacking leucine and tryptophan (SD-LW) and were replica-plated onto various SD selection media, including media lacking leucine, tryptophan, and histidine (SD-LWH, +5 mM 3-AT) and media lacking leucine, tryptophan, and adenine (SD-LWA).

## Results

### Identification of 17 *OsANTH* Genes in Rice

**FIGURE 1:**
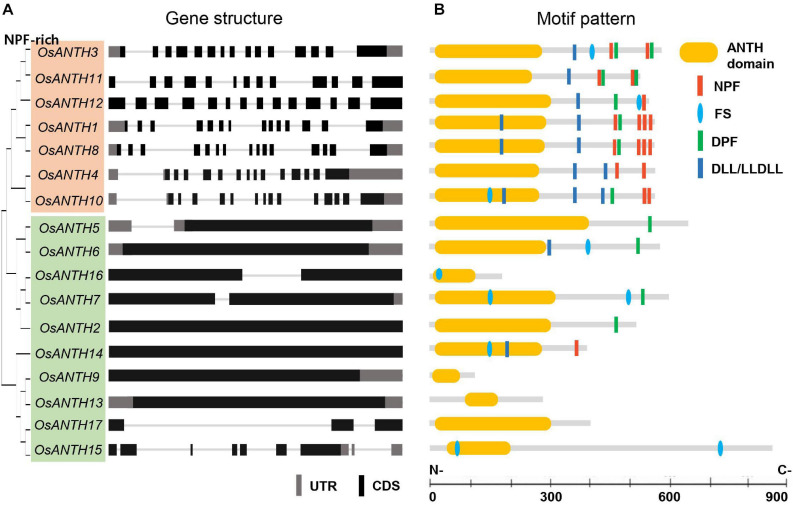
Gene structure and motif pattern of rice AP180 N-terminal homology (ANTH) proteins. **(A)** Phylogenetic tree and gene structure of seventeen *ANTH* genes. Upper 7 and lower 10 ANTH proteins are divided into two subgroups according to gene structure (whether they contain no or only a few introns or many introns). Black and gray boxes indicate coding sequences (CDSs) and untranslated regions (UTR), respectively, and lines represent introns. **(B)** Motif analysis of ANTH proteins: ANTH, asparagine-proline-phenylalanine (NPF), the clathrin-binding motif aspartic acid-leucine-leucine/leucine-leucine-aspartic acid-leucine-leucine (DLL/LLDLL), the clathrin-binding motif phenylalanine-Serine (FS), and the α-adaptin-binding motif aspartic acid-proline-phenylalanine (DPF). Bar in each section represents 100 aa.

To obtain all ANTH domain-containing sequences in rice, we reannotated protein sequences from the Rice Genome Annotation Project^1^ on the Pfam database (PF07651.9). A total of 17 genes were identified as putative *ANTH* genes in rice and named *OsANTH1* to *OsANTH17* based on their physical location on the chromosomes (Supplementary Table 1). To further understand the structure of the *OsANTH* genes, we compared the corresponding genomic DNA sequences and obtained the exon and intron structures of the *OsANTH* genes (Figure 1A). Intron numbers varied from 0 to 15.

All candidate OsANTH proteins were analyzed using SMART and Pfam tools, indicating that they all possessed the ANTH domain at the N-terminal region of the protein and exhibited various protein sizes (Figure 1B). OsANTH9 was the smallest protein with 116 amino acids, and OsANTH15 was the largest protein with 863 amino acids.

### Phylogenetic Tree and Motif Composition Analyses of OsANTH Proteins

**FIGURE 2:**
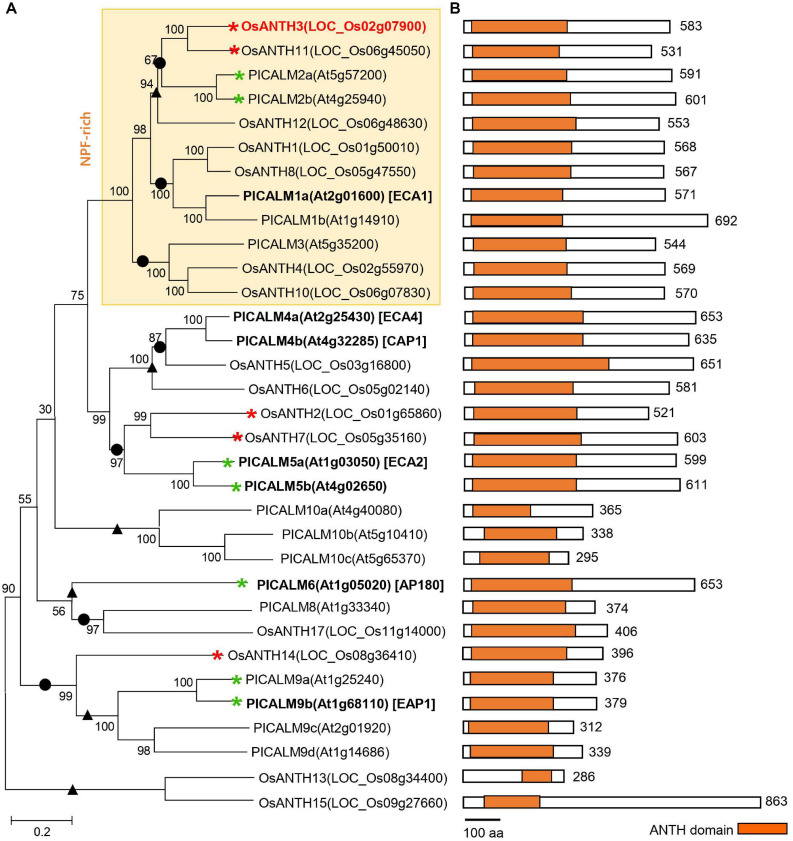
Phylogenetic analysis of the AP180 N-terminal homology (ANTH) family from *Oryza sativa* and *Arabidopsis thaliana*. **(A)** Phylogenetic tree constructed using the protein sequences for each gene with MEGA7 software. Red and green asterisks indicate *ANTH* genes with pollen-preferential expression in rice (in this study) and *Arabidopsis* (Muro et al., 2018; Supplementary Figure 6), respectively. Bold letters indicate the functionally characterized genes, and red color indicates genes for which the mutant phenotype was analyzed in this study. We used 1,000 bootstrap replicates, and the number at each node is a bootstrap value. • and ▲ indicate branches conserved and diversified between *Arabidopsis* and rice, respectively. **(B)** Schematic diagram of the ANTH protein primary structure with the ANTH domain. The length of each ANTH protein is shown on the right regarding the number of amino acids (aa). The scale bar indicates 100 aa. Orange boxes indicate the position and length of the conserved ANTH domain in the family. Each domain is presented in the N-terminal of the protein. *OsANTH9* and *OsANTH16* are not aligned because the gene length is too short.

Metazoan and fungal genomes have fewer ANTH proteins than plants, implying that the ANTH proteins in plants have evolved more divergent functions than those in non-plant systems (Zouhar and Sauer, 2014). To understand the evolutionary relationships of ANTH proteins in plant species, the amino acid sequences of 17 ANTH proteins in rice and 18 ANTH proteins in *Arabidopsis* (PICALMs) were isolated and aligned (Figure 2A). The ANTH families of rice and *Arabidopsis* are composed of a similar number of family members, which seems to be due to similar evolutional progress. *Arabidopsis* and rice members are not clustered according to species, and instead, many members seem to have coevolved into monocots and dicots and duplicated. However, some members seem to be restricted to either *Arabidopsis* or rice. For example, OsANTH13 and OsANTH15 only exist in rice, while three PICALM10 homologs do not exist in rice (Figure 2A).

Mammalian ANTH proteins are divided into subfamily groups depending on the presence of actin-binding motifs. However, plant ANTH proteins do not have an I/LWEQ sequence, which is an actin-binding motif. Instead, they are divided into two subgroups based on the presence of asparagine-proline-phenylalanine (NPF) motifs: the NPF-rich and NPF-less subfamilies (Holstein and Oliviusson, 2005; Zouhar and Sauer, 2014). NPF motifs bind to Eps15 homology (EH) domain-containing proteins that function as regulators of endocytosis through their ability to interact with other proteins involved in CME. Our analysis demonstrated that the NPF-rich subfamily had 14–15 exons with a corresponding number of introns, while the NPF-less subfamily had few or no introns in rice (Figure 1A). In addition, the exon-intron structures of the closely linked genes in the phylogenetic tree were almost identical.

Plant ANTH proteins contain variations of the conserved mammalian ANTH signature motif (Ford et al., 2001), namely KAT(X)5/6P(X)3K/RH/Y, which enables ANTH proteins to bind to the PM. The ANTH families of both rice and *Arabidopsis* have ANTH domains in the N-terminal region (Figure 2B). However, OsANTH9, OsANTH13, OsANTH15, and OsANTH16 contained shorter ANTH domains than the other family members (Figure 1B), and the ANTH consensus region was not well conserved with other OsANTH protein sequences. Using the rice male gamete expression database (RMEDB^7^) to check the expression, these putative *OsANTH* genes were rarely expressed and may be pseudogenes that lost their function during evolution (Supplementary Figure 3). Protein alignment analysis of 13 *OsANTH* genes, excluding the abovementioned 4 *OsANTH* genes, showed that the ANTH signature motif is well conserved between the α-helix 1 and α-helix 2 motifs, except for partial conservation in OsANTH11 and OsANTH14 (Supplementary Figure 2).

Relatively, the C-terminal sequences of most ANTH domain-containing proteins are diversified (Supplementary Figure 2) and are known to interact with other components of the vesicle-generating machinery, such as clathrin (Legendre-Guillemin et al., 2004; de Craene et al., 2012). In this respect, ANTH domain-containing proteins act as a bridge between PIP_2_ and several components of the CME machinery. Endocytosis is a complex molecular process that depends on regulated interactions between a variety of proteins and lipids through specific modules. Excluding OsANTH9, OsANTH13, and OsANTH17, the C-terminal of the remaining 14 ANTH proteins possessed binding sites for various proteins including EH, clathrin, and the EAR-binding domain. The presence of the mammalian clathrin-binding motifs DDL and FS, as well as the α-adaptin-binding motif DPF (Brett et al., 2002), within the OsANTH protein sequences (Figure 1B) indicated conserved motifs of ANTH in plants and animals. In contrast to their yeast and mammalian counterparts, OsANTH proteins do not contain conserved ubiquitin-interacting motifs, such as the DPW and FXDXF motifs or the I/LWEQ motif, which are required for interaction with ubiquitylated proteins using the α-adaptin of AP-2 or the actin cytoskeleton, respectively (Holstein and Oliviusson, 2005).

### Expression Pattern Analysis of *OsANTH* Genes

**FIGURE 3:**
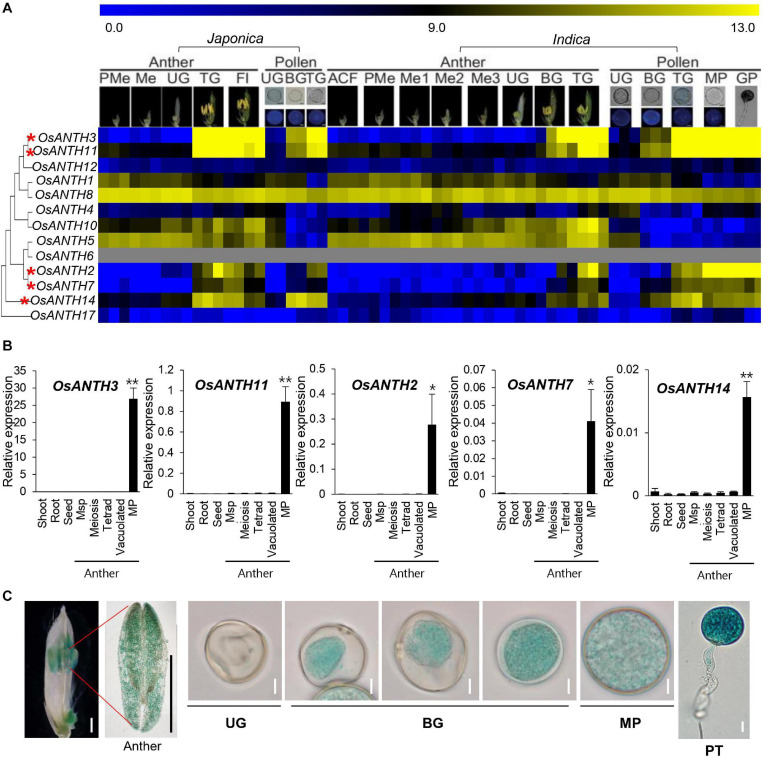
Expression patterns of *OsANTH* genes in rice. **(A)** Heatmap showing expression levels as determined by microarray analysis. Yellow color in the heatmap indicates a high level of expression, whereas dark blue indicates low expression. Numeric values indicate an average of the normalized log_2_ intensity value of the microarray data. Pollen-preferentially expressed *OsANTH* genes are indicated by red asterisks. ACF, archesporial cell-forming stage; BG, bi-cellular gametophyte stage; Fl, flowering stage; GP, germinated pollen; Me, meiotic stage; Me1, meiotic leptotene stage; Me2, meiotic zygotene-pachytene stage; Me3, meiotic diplotene-tetrad stage; MP, mature pollen stage; PMe, pre-meiosis; TG, tricellular pollen stage; UG, uni-cellular gametophyte stage. **(B)** Expression of pollen-preferred ANTH domain-containing protein-coding genes in *Oryza sativa* (*OsANTH* genes) analyzed via qPCR in various tissues of rice. Msp, Microspore; MP, mature pollen. Rice ubiquitin 5 (*OsUbi5*, *LOC_Os01g22490*) was used as an internal control. The *y*-axis shows the expression level relative to *OsUbi5*, while the *x*-axis shows the samples used for analyses. Error bars represent the standard errors of three biological replicates. Significant differences are indicated by asterisks; *p < 0.01 and **p < 0.0001. Data were analyzed by employing one-way analysis of variance with repeated measures using Tukey’s pairwise comparison test. **(C)** Expression pattern of OsANTH3-GUS in transgenic rice plants at the anther and sequential stages of pollen development. UG, unicellular gametophyte stage; BG, bicellular gametophyte stage; MP, mature pollen stage. Bar in flower and anther image = 1 mm; bar in pollen image = 10 μm.

To identify possible ANTH candidates related to pollen tube growth in rice, we used the RMEDB database, which provides meta-expression analysis focused on rice male gamete development (Chandran et al., 2020). The meta-expression data in the context of the phylogenetic tree were produced to check the function of *OsANTH* genes with respect to anther and pollen development compared with other tissues/organs (Figure 3A). In addition, we examined their expression profiles in RNA-sequencing samples (Supplementary Figure 3; Hong et al., 2020). We found that the expression patterns of five genes (*OsANTH3, OsANTH11, OsANTH7, OsANTH2*, and *OsANTH14*) were closely associated with late pollen development, i.e., at the tricellular pollen grains, mature pollen, and germinated pollen stages. *OsANTH3, OsANTH11, OsANTH2*, and *OsANTH7* had similar expression patterns. Especially, *OsANTH3* and *OsANTH11* had high expression in the late pollen development stage and were located in the same sister node of the phylogenetic tree. Conversely, *OsANTH8*, which was in the same sister node as *OsANTH3* and *OsANTH11*, showed high expression in not only late pollen development but also in somatic tissues as well. Therefore, *OsANTH8* is expected to have a role in various tissues including pollen and somatic tissues, while five *OsANTH* genes are expected to be specific to pollen function.

Further, to verify the meta-expression data, we performed qRT-PCR using eight tissues: shoot, root, seed, anthers containing meiosis microspore, tetrad microspore, young microspore, vacuolated pollen, and mature pollen (Figure 3B). Similar to the microarray results, *OsANTH3, OsANTH11, OsANTH7, OsANTH2*, and *OsANTH14* showed preferential expression in the mature pollen-containing anther but were not significantly expressed in other tissues. Therefore, these five genes (*OsANTH3, OsANTH11, OsANTH7, OsANTH2*, and *OsANTH14*) may play key roles in pollen tube growth and other processes that occur after pollen maturation. *OsANTH3* and *OsANTH11* belong to the NPF-rich group, while *OsANTH7*, *OsANTH2*, and *OsANTH14* belong to the NPF-less group (Figure 1B). *OsANTH3* and *OsANTH11*, which belong to the NPF-rich group, have a similar expression pattern. These two genes were duplicated since the divergence of *Arabidopsis* and may be functionally redundant. Especially, *OsANTH3* showed the highest level of expression among the five genes in the mature pollen.

To investigate the *in planta* expression of the *OsANTH3* gene in rice, the expression of GUS under the control of the *OsANTH3* promoter was investigated (Figure 3C). GUS expression appeared in the pollen of the anthers. In particular, *OsANTH3* showed peak expression in the late pollen stages, including in mature pollen and germinating pollen, after initial expression in the bicellular pollen stage (Figure 3C). In germinated pollen, GUS was expressed in the pollen tube as well as the pollen grain. It can be expected that *OsANTH3* may play a role in pollen germination or pollen tube elongation.

### Subcellular Localization of Four ANTH Proteins

**FIGURE 4:**
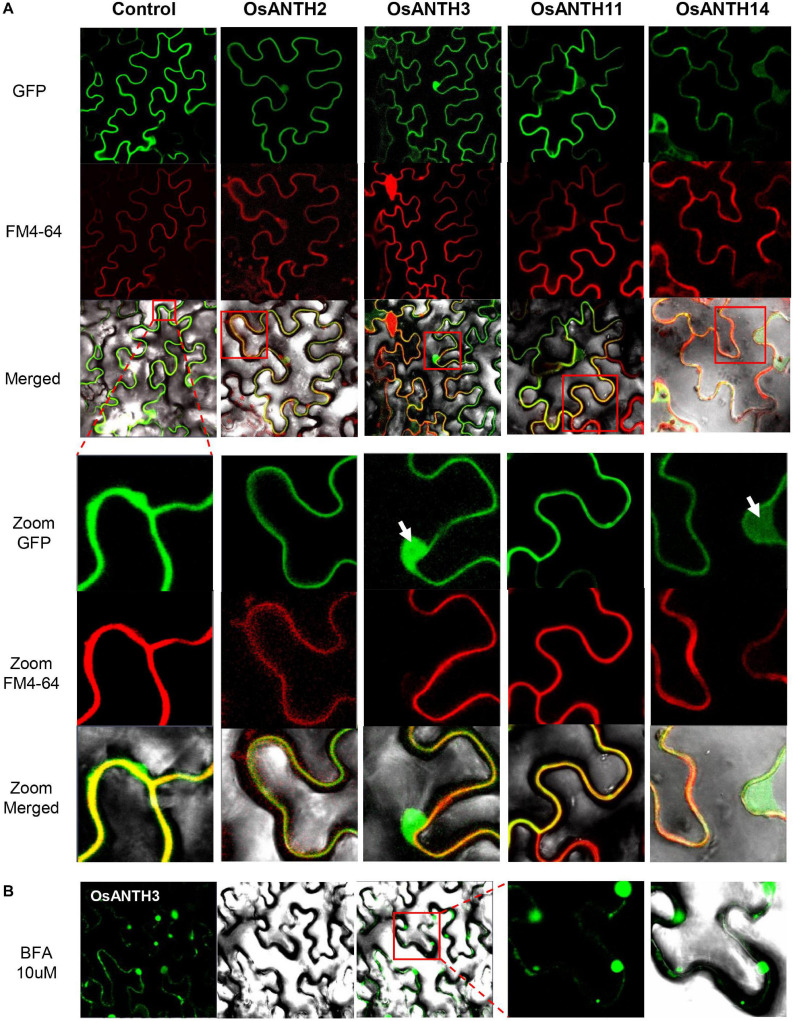
Subcellular localization of *Oryza satvia* AP180 N-terminal homology domain-containing (OsANTH) proteins in tobacco leaves using laser scanning confocal fluorescence microscopy. **(A)** Upper panel shows green fluorescence in tobacco epidermal cells in leaves infiltrated with four OsANTH-green fluorescence protein (GFP) fusion proteins, the center panel shows the same leaves stained with FM4–64, and the lower panel shows the merged images from GFP, FM4-64, and bright field. The focused image is the plasma membrane (PM) and shows an enlarged portion of the red box displayed in the merged pictures. Yellow signals show a merged image from the OsANTH localization and PM signal. The white arrows indicate the signal not merged with the PM (OsANTH3-nucleus, OsANTH14-cytoplasm). **(B)** The first panel shows green fluorescence in tobacco epidermal cells of leaves infiltrated with OsANTH3-GFP fusion proteins when treated with 10 μM BFA. The second panel shows the images in bright field. The third panel shows the merged images from GFP and bright field. The last two panels are GFP and merged images that enlarge the red box in the third panel.

ANTH proteins are known to assemble clathrin to the PM to initiate CME (Kaneda et al., 2019). In *Arabidopsis*, *PICALM5a, PICALM5b*, and *PICALM6* have been shown to localize apical PM and the cytosol of pollen tubes (Barth and Holstein, 2004; Muro et al., 2018). To evaluate whether the OsANTH proteins are also localized at the PM, we constructed GFP-fused OsANTH proteins driven by the CaMV 35S promoter and examined their subcellular localization in *Agrobacterium*-infiltrated tobacco epidermal cells. The experiment was conducted with four pollen-preferred genes (*OsANTH3, OsANTH11, OsANTH2*, and *OsANTH14*), and all four genes were found to have GFP signals in the nucleus, PM, and cytosol (Figure 4A). When tobacco cells were stained with FM4-64, which is a marker for membrane trafficking in plant cells (Bolte et al., 2004; Wang et al., 2005), the signal from all four OsANTH-GFP proteins overlapped with the signal from the FM4-64 stain. These results suggest that four OsANTH proteins with pollen preferential expression are localized in the PM and internalized through endocytosis (Nagawa et al., 2012) when transiently expressed in tobacco leaves.

BFA is a fungal metabolite that inhibits exocytosis but allows the first step of endocytosis to proceed in eukaryotic cells (Lippincott-Schwartz et al., 1991; Baluška et al., 2002; Nebenführ et al., 2002; Geldner et al., 2003). Thus, BFA inhibits the endocytic recycling of PM proteins. When BFA is used to treat plant cells, PM proteins or peripheral membrane proteins that are rapidly recycled, are internalized via endocytosis and gather in BFA-induced compartments to form an endosome. Due to these characteristics, BFA has been an important tool for endocytosis research in *Arabidopsis* (Samaj et al., 2004). We treated the tobacco leaves expressing OsANTH3-GFP with 10 μM BFA to determine whether OsANTH was involved in endocytosis. One hour later, we were able to observe several endosomal vesicles in the cytoplasm close to the PM (Figure 4B), while the BFA bodies were not observed with OsANTH11-GFP expression.

### Genetic Study Using the Mutation of *OsANTH3*

**FIGURE 5:**
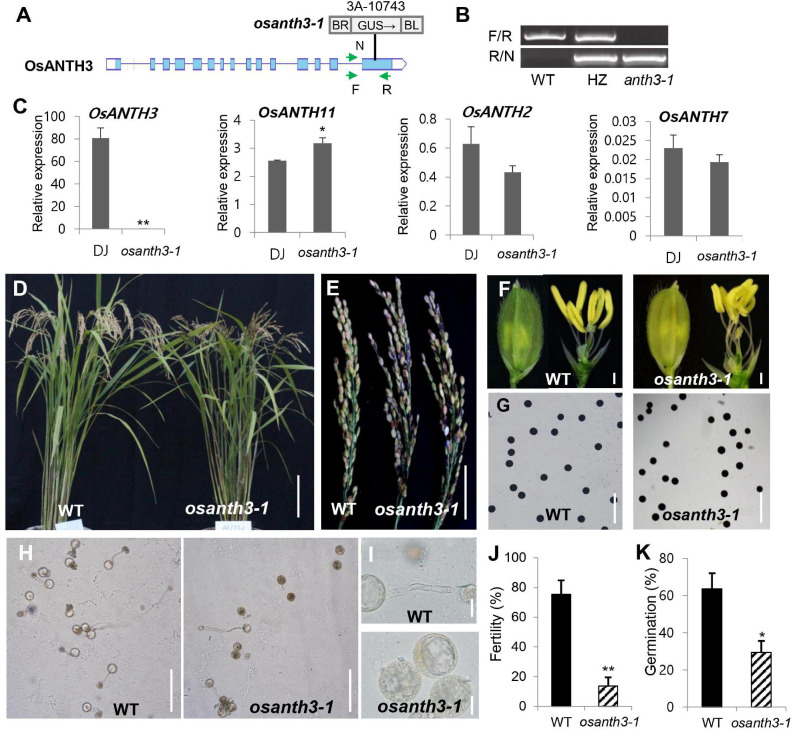
Phenotypes of plants containing mutations in AP180 N-terminal homology (ANTH) domain-containing protein-coding gene 3 in *Oryza sativa* (*OsANTH3*) (*LOC_Os02g07900*). **(A)** Schematic diagram of the *osanth3-1* mutant with T-DNA insertion. **(B)** Polymerase chain reaction (PCR) analysis for genotyping using two primer sets (F/R and R/N). Genotyping results showed that *osanth3-1* was homozygous. **(C)** qRT-PCR for four genes (*OsANTH3, OsANTH11, OsANTH2*, and *OsANTH7*) in Dongjin (WT) and *osanth3-1*. Rice ubiquitin 5 (*OsUbi5*, *LOC_Os01g22490*) was used as an internal control. The *y*-axis shows the expression level relative to *OsUbi5*, while the *x*-axis shows the samples used for analyses. Error bars represent the standard errors of three biological replicates. **(D)** Vegetative growth was not significantly different between the WT and *osanth3-1*, but *osanth3-1* shows partial male sterility. **(E)** Panicle of the *osanth3-1* mutant observed with many empty grains due to poor germination. **(F)** Whole spikelet (left side) and spikelet after removing lemma and palea (right side) of *osanth3-1* are similar to those of the WT. **(G)** Pollen grains of the WT and *osanth3-1* mutant stained with KI solution exhibited normal starch accumulation. **(H,I)** Microscopic photograph of pollen germination in the WT and *osanth3-1* mutant. Compared with that in the WT, *osanth3-1* exhibited a lower germination percentage **(J)**. Fertility rate was calculated as (average of the number of seeds normally germinated)/(total seeds) × 100 for each panicle. **(K)** The germination rate was calculated as (the average of the number of germinated pollen)/(total pollen) × 100, *n* = 100. Significant differences are indicated by asterisks; *p < 0.01 and **p < 0.0001. Scale bars, 10 cm in **(D)**; 5 cm in **(E)**; 10 μm in **(F)**; and 20 μm in **(I)**; Bars = 200 μm in **(G,H)**. Error bars represent the standard errors of three biological replicates **(C,J)**.

To investigate the possible role of OsANTH protein in pollen, we decided to focus on *OsANTH3* as it was preferentially expressed, and expressed at a high level, in pollen. We identified a T-DNA mutant in *OsANTH3* in the PFG library and confirmed the line contained a single insert in the 15th exon of OsANTH3 by genotyping (Figure 5A). After isolating the homozygous mutant (Figure 5B), we observed about an 80% reduced fertility rate in the T-DNA insertional homozygous mutant without other obvious growth defects (Figure 5J). To confirm the defect of *OsANTH3* in *osanth3-1*, we performed qRT-PCR analysis on mature anthers of *osanth3-1* and the WT (Figure 5C). Compared with the high expression in WT, we found that *OsANTH3* expression was significantly reduced, confirming that the T-DNA insertional mutant has a defect in the function of *OsANTH3*. We then analyzed the expression of other pollen-preferred *OsANTH* genes in the mutant. *OsANTH2* and *OsANTH7* expression slightly but not significantly decreased in the mutant, while expression of *OsANTH11* slightly increased, suggesting that *OsANTH2* and *OsANTH7* were not affected by *OsANTH3*; however, *OsANTH11* could partially compensate for the defect in the mutant.

To find the cause of the partial sterile phenotype of the *osanth3-1* homozygous mutant (Figures 5D,E), we further examined phenotypical changes. Mutant plants produce normal panicle (Figure 5F), floral organs, and pollen grains (Figure 5G). However, we found that the germination percentage of pollen in *osanth3-1* was 29.38%, compared with 63.67% in the WT (Figures 5H,K). The KI-staining results showed no difference between the WT and mutant pollens for maturation (Figure 5G), indicating that that the reduction in the percentage of germination was not caused by a lack of starch in the pollen grain. The size variation of pollen grains indicates the hydration status of rice pollen grains, indicating some smaller grain are not hydrated fully. Although the germination percentage in *osanth3-1* pollen was more reduced than that in WT pollen, germinated *osanth3-1* pollen normally grows pollen tubes (Figures 5H,I). The homozygous mutant generated by CRISPR-Cas9, named *osanth3-2*, also showed a similar phenotype (Supplementary Figure 5), confirming the role of OsANTH3 in pollen germination. Our study reveals that OsANTH3 plays a role in rice pollen germination.

**FIGURE 6:**
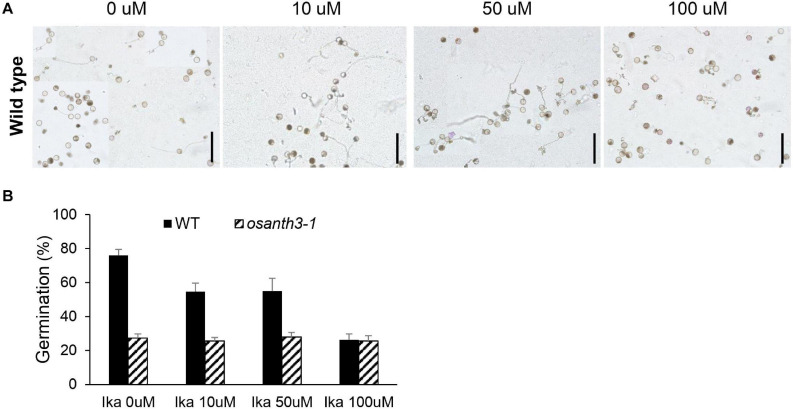
Rice pollen germination percentage and clathrin-mediated endocytosis patterns under Ikarugamycin (IKA) treatment at varying concentrations. **(A)** Images of rice pollen placed in germination media with different concentrations of IKA (0, 10, 50, and 100 μM). All media contained 0.2% dimethyl sulfoxide (DMSO). Bars = 200 μm. **(B)** Germination percentage of the wild-type and *osanth3-1* plants at different IKA concentrations. Germination rate was calculated with three replicates (each of the replicates was calculated for *n* > 100).

To further examine how CME affects germination, we treated a CME inhibitor, IKA. As the concentration of IKA increased from 0 to 100 μM (Figure 6A), the germination percentage decreased, suggesting that CME can affect rice pollen germination. Compared with the WT, *osanth3-1*, which does not normally perform CME due to a lack of ANTH3 function, showed a low germination percentage regardless of IKA concentration (Figure 6B). The results suggest that CME affects rice pollen germination and is mediated by OsANTH3.

## Discussion

ANTH proteins act as adaptor proteins for CME to recycle or degrade cargo proteins in the membrane. In this study, we explored the physiological role of CME and *ANTH* genes regarding pollen germination in rice. We found 17 *OsANTH* genes in the rice genome; four genes contained a partial ANTH domain and were expressed at undetectable levels.

ANTH proteins contained a conserved PIP_2_-binding site in the α-helix 2 and α-helix 3 motifs at the N-terminal region (Supplementary Figure 2), due to which the proteins bind to the PM (Holstein and Oliviusson, 2005). Their secondary structure in the N-terminal region was also very similar (Supplementary Figure 6). The C-terminal region of OsANTH proteins, which contains various of motifs, was predicted to be exposed to the cytoplasm, as a general feature of the ANTH protein family. Therefore, this region may be able to induce the recruitment of coat component and clathrin assembly. When expressed in tobacco leaves, signals from the four GFP-OsANTH fusion proteins (*OsANTH2, OsANTH3, OsANTH11*, and *OsANTH14*) with pollen preferential expression were observed in the PM, cytosol, and nucleus (Figure 4). Similar to our results, the ANTH proteins in *Arabidopsis* (PICALM5a and PICALM5b) are localized in mature pollen grains and the cytoplasm and PM of germinated pollen tubes (Muro et al., 2018). Localization of ANTH proteins in the nucleus has not yet been reported in plants; however, in animals, the epsin protein has been reported to be translocated from the cytoplasm to the nucleus, suggesting that it may convey signals from the endocytic pathway to the transcription machinery (Hurtley, 2000).

Members of the ANTH family are also found in most taxa, excluding the Chromista and Excavata. The Plantae ANTH family has evolved to be more subdivided than other kingdoms (Opisthokonta, Amoebozozoa, and Euglenozoa). Plantae, which undergo endocytosis and endosomal sorting, all had a complex evolution, as illustrated by the high number of duplications in each subfamily. In most plants, the molecular mechanisms and how this differs in different tissues are not well characterized. However, this increased number of *ANTH* genes suggests that endocytosis and endosomal functionality might be specialized to different cell types or cargoes, and these genes have different function at different steps of trafficking in Plantae (de Craene et al., 2012). Even with close evolutionary relationships, each gene might have unique roles in each species (Zouhar and Sauer, 2014) or can be responsible for specified endocytic functions in different tissues. It has been reported that nine ANTH member homologs exist in monocots and dicots, two members are specific to dicots, and seven members are specific to the Brassica family (Zouhar and Sauer, 2014). It is noteworthy that some members seem to have coevolved in monocots and dicots for pollen function. For example, pollen-expressed OsANTH3 and OsANTH11 are homologous to PICALM2a and PICALM2b in *Arabidopsis*, which are highly expressed in pollen (Supplementary Figure 4). Until now, there have been few reports on the function of *ANTH* genes in other species besides *Arabidopsis*. Therefore, further studies are required to determine whether pollen-preferred *ANTH* genes function as different adaptors during pollen germination.

ANTH proteins can be divided into two subgroups, and the C-terminal region of the NPF-rich subgroup was more conserved than that of the NPF-less subgroup (Figure 1). *OsANTH3* and *OsANTH11* genes belonging to the NPF-rich subgroup were pollen-preferred genes (Figure 1). Few studies have been conducted on the NPF-rich groups in *Arabidopsis*, and all the genes studied in relation to pollen germination and tube growth belonged to NPF-less groups.

**FIGURE 7:**
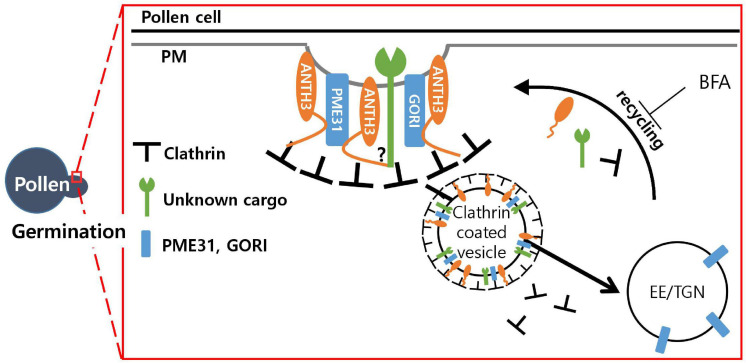
Model of the possible role of *Oryza satvia* AP180 N-terminal homology domain-containing protein 3 (OsANTH3) in clathrin-mediated endocytosis in germinating pollen. *OsANTH3* might function as an adaptor for clathrin-mediated endocytosis, which is critical for rice pollen germination. Unknown cargo and/or GORI and PME31 proteins might be endocytosed from the plasma membrane (PM) to the early endosome/trans-Golgi network (EE/TGN) by interacting with the N-terminal of the OsANTH3 protein. Brefeldin A (BFA) treatment inhibits recycling from the EE/TGN to the PM, and OsANTH3 can be formed as BFA bodies in the cytoplasm.

Like the ANTH proteins in other plant species, OsANTH proteins lack the DPW and FXDXF motifs, which are present in animals ANTH protein for α-adaptin binding (Owen et al., 1999; Brett et al., 2002). Instead, the NPF and DPF motifs can recruit EH domain-containing proteins, bind α-adaptin, and function as clathrin-binding motifs (Scheele et al., 2001). This suggests that NPF-rich OsANTH3 not only combine with α-adaptin to participate in CCV but also function as a clathrin-binding motif. The main endocytic clathrin adapter AP-2 alone is not sufficient to generate CCVs (Brod et al., 2020). CALM and AP180, the animals ANTH proteins, are abundant in CCVs similar to AP-2 (Prasad and Lippoldt, 1988; Skruzny et al., 2015). Additionally, it has been proposed that ANTH proteins act as specific monomeric cargo adapters that recruit cargo to the CCV nucleation site independent of heterotetrameric AP-2 (Holstein and Oliviusson, 2005). For example, AP180 regulates synaptic vesicle size and was shown to regulate the abundance of glucose-dependent insulinotropic polypeptide 1 receptors at postsynaptic elements in *Caenorhabditis elegans*, and disabled-2 targets low-density lipoprotein receptors for endocytosis (Miller and Lefkowitz, 2001; Burbea et al., 2002; Mishra et al., 2002). Furthermore, huntingtin-interacting protein 1 seems to function as an adaptor for a glutamate receptor in animals (Metzler et al., 2007), and PICALM5 in *Arabidopsis* proteins serves as a specific loading adaptor to recycle ANXUR proteins (Muro et al., 2018). In our motif analysis, all seven OsANTH proteins belonging to the NPF-rich group had DLL motifs in the same region, suggesting that they can function in regular endocytic CCVs. Although whether OsANTH is the only protein that assembles clathrin and how it interacts with binding proteins still need to be verified, this suggests that OsANTH is an important factor as a monomeric cargo-specific adaptor for CME (Figure 7).

The TGN in plants is a major sorting system and receives recycled material from endocytosis (Rosquete et al., 2018). The TGN functions as the EE in plant cells (Bolte et al., 2004; Dettmer et al., 2006; Lam et al., 2007). The inhibitor BFA affects membrane traffic in animal secretory and endocytotic pathways (Lippincott-Schwartz et al., 1991). In plants, large intracellular compartments are formed in response to BFA treatment. BFA inhibits the recycling of PM proteins by interfering with secretion from the TGN/EE to the PM in intracellular traffic between them, creating BFA compartments (Lam et al., 2009). In the localization experiment observed on tobacco leaves using the GFP-fusion protein, several endosomal vesicles called BFA bodies were observed around the cytoplasm close to the PM. These results indicate that OsANTH3 prefers to circulate material from the PM to the TGN/EE. In particular, it is assumed that OsANTH3 plays a role in recirculating the signal protein or extracellular material present in the PM, either through endocytosis or by embedding in the PM itself. Integral PM proteins to be internalized via CME are marked for uptake by signal peptides and posttranslational modification like ubiquitination. In plants, it is still unknown how cargo recycles or degrades. Clathrin-independent endocytosis (CIE) appears to be restricted to the tip apex, while CME preferentially occurs in the shoulder region of the apex (Dhonukshe et al., 2007; Moscatelli et al., 2007; Moscatelli, 2008; Zhao et al., 2010). IKA, an inhibitor of CME in animal cells, has been used to dissect the endocytic pathway (Hasumi et al., 1992; Luo et al., 2001). In fact, there are reports relating to endocytosis in which the length of pollen tubes was reduced when IKA was used to treat tobacco pollen tubes (Moscatelli et al., 2007). Our results showed that the higher the concentration of IKA on the pollen, the lower the gemination rate, but the pollen tube length of the germinated pollen was found to be normal. This can be explained through the observation of tobacco pollen tubes in response to the IKA treatment; when IKA was processed, endocytosis was not completely inhibited, and the pollen tube gradually reached the normal length. Therefore, even if CME is inhibited, pollen tube growth is possible through CIE. This may be because rice has a relatively short pollen tube length and growth time.

Animal and yeast cells internalize preferential signal molecules through CME in a variety of signal transduction pathways (Goldstein et al., 1985; Wendland et al., 1999). Several studies on *Arabidopsis* have demonstrated that auxin flow is partially mediated by membrane trafficking-dependent intracellular relocalization and/or degradation of PIN auxin efflux transporters (Kleine-Vehn et al., 2006). In fungus, the CME pathway may be important during germination, germ-tube growth, and initiation of appressorium formation (Atkinson et al., 2002). In plants, most endocytic mechanisms are mediated by the coat protein clathrin. In addition, it has been proven that rapid activation of endocentric processes occurs during the seed germination (Pagnussat et al., 2012). In our study, for the WT under CME-inhibiting conditions, pollen germination was reduced, but not tube elongation. In addition, the results of *osanth3-1* showed a reduction in the germination percentage; however, the growth of the pollen tube was normal. This low generation percentage in *osanth3-1* eventually led to a decrease in fertility. As a result of drawing the network to find the candidate genes involved in germination signaling using endocytosis and OsANTH3 (Supplementary Figure 7), it was possible to suggest that ANTH could interact with various signaling proteins, including receptor or membrane-integrated proteins involved in endocytosis.

In the Y2H screening for the identification of direct protein interactions (Supplementary Figure 7), the C-terminal of OsANTH3 (1096∼end) was found to interact with the PME31 (pectin methylesterase) (Kim Y. et al., 2020) and GORI proteins (Kim et al., 2021), which are both important for rice pollen germination. GORI encodes a seven WD40 motif protein that is homologous to REN4 in *Arabidopsis*. Knock-out of *GORI* caused 20% pollen germination percentage but germinated pollen has defect on tube elongation as well, causing full male sterility (Kim et al., 2021). REN4 was shown to interact with PICALM9b/EAP1 (Li et al., 2018), similarly to interactions between GORI and OsANTH3. The WD40 motif might function as a scaffold to form an endocytosis complex. Taken together, we were able to draw a working model of OsANTH in a pollen (Figure 7). We still need to further confirm how this complex affects germination signaling; however, we assume that OsANTH proteins are involved in CME by interacting with various membrane-bound proteins or signaling molecules through the C-terminal to form a functional complex.

## Data Availability Statement

The datasets presented in this study can be found in online repositories. The names of the repository/repositories and accession number(s) can be found in the article/Supplementary Material.

## Author Contributions

SL, K-HJ, and Y-JK conceived and designed the experiments, analyzed data, and wrote the manuscript. W-JH, JS, and E-JK contributed the phenotypical and computational analysis. SP contributed the research design. All authors contributed to the article and approved the submitted version.

## Conflict of Interest

The authors declare that the research was conducted in the absence of any commercial or financial relationships that could be construed as a potential conflict of interest.

## Footnotes

1 http://rice.plantbiology.msu.edu/

2 https://www.ncbi.nlm.nih.gov/

3 https://phytozome.jgi.doe.gov/pz/portal.html

4 http://www.ncbi.nlm.nih.gov/geo/

5 https://genevestigator.com/gv/

6 http://mev.tm4.org/

7 http://ricephylogenomics-khu.org/RMEDB/home.php
