# Supplementary material for: Global Identification of ANTH Genes Involved in Rice Pollen Germination and Functional Characterization of a Key Member, OsANTH3
Source: Front Plant Sci. 2021 Apr 13;12:609473. doi: 10.3389/fpls.2021.609473 (PMC8076639; doi:10.3389/fpls.2021.609473)
Supplement: Supplementary file 1 [file Data_Sheet_1.pdf]

Additional data

Table S1. Accession numbers of *ANTH* genes

Gene	Locus	Gene	Locus
OsANTH1	LOC_Os01g50010	*PICALM1a*	Loc_At2g01600
OsANTH2	LOC_Os01g65860	*PICALM1b*	Loc_At1g14910
OsANTH3	LOC_Os02g07900	*PICALM2a*	Loc_At5g57200
OsANTH4	LOC_Os02g55970	*PICALM2b*	Loc_At4g25940
OsANTH5	LOC_Os03g16800	*PICALM3*	Loc_At5g35200
OsANTH6	LOC_Os05g02140	*PICALM4a*	Loc_At2g25430
OsANTH7	LOC_Os05g35160	*PICALM4b*	Loc_At4g32285
OsANTH8	LOC_Os05g47550	*PICALM5a*	Loc_At1g03050
OsANTH9	LOC_Os06g06640	*PICALM5b*	Loc_At4g02650
OsANTH10	LOC_Os06g07830	*PICALM6*	Loc_At1g05020
OsANTH11	LOC_Os06g45050	*PICALM8*	Loc_At1g33340
OsANTH12	LOC_Os06g48630	*PICALM9a*	Loc_At1g25240
OsANTH13	LOC_Os08g34400	*PICALM9b*	Loc_At1g68110
OsANTH14	LOC_Os08g36410	*PICALM9c*	Loc_At2g01920
OsANTH15	LOC_Os09g27660	*PICALM9d*	Loc_At1g14686
OsANTH16	LOC_Os09g29680	*PICALM10a*	Loc_At4g40080
OsANTH17	LOC_Os11g14000	*PICALM10b*	Loc_At5g10410
		PICALM10c	Loc_At5g65370

Table S2. Os*ANTH gene* isogene-specific primers for qPCR and pGREEN cloning.

Genes	Sense Primers	Antisense Primers
**qPCR**		
OsANTH2	5'-ATCACCGACGGGAGTTACGA-3'	5'-CCGATGAACCGCTGGAGTAT-3'
OsANTH3	5'-AGGCATCAACTTCCCTGGAC-3'	5'-CGTCTCGTCTTCGTAGAGGC-3'
OsANTH7	5'-AGCGAGATGACGCTGGAG-3'	5'-GTACAGCGACACCGACACC-3'
OsANTH11	5'-AGAGCCGGTACCACTAGCAA-3'	5'-AAGCTGAGGCCTTGTTTTCA-3'
OsANTH14	5'-GTACTTCGAGCTTTGCCGGA-3'	5'-GCCTTCTCCTCGCTCACC-3'
**pGREEN cloning**		
OsANTH2	5'-GACGGTATCGATAAGCTT ATGGCGCCGAGCAAGCT-3'	5'- AGGAATTCGATATCAAGCTTCAACAATGA ACTGTAACTCC-3'
OsANTH3	5'-GACGGTATCGATAAGCTT ATGGCTGGGGGTGGAACGA-3'	5'- AGGAATTCGATATCAAGCTTCATCATGCCT GTCCCAGAGT-3'
OsANTH4	5'- GACGGTATCGATAAGCTTATGACGA CCGCGCGGCAAT-3'	5'- AGGAATTCGATATCAAGCTTAACTAGCAA GTTCCCGCTCC-3'
OsANTH14	5'- CGGTATCGATAAGCTTATGACGACC GCGCGGCAAT-3'	5'- GGAATTCGATATCAAGCTTAACTAGCAAGT TCCCGCTC-3'
**Yeast vector cloning**		
ANTH3-C terminal	5'- GCCATGGAGGCCAGTGAATTCGAA GAGTACATCCGAGAA-3'	5'- AGCTCGAGCTCGATGGATCCTCATATCAAA CTTGAATGTC-3'
ANTH3-N terminal	5'- GCCATGGAGGCCAGTGAATTCGGG TCGTGGCGGCGCAA-3'	5'-AGCTCGAGCTCGATGGATCC TCCAGTGGTTTCTGGTGG-3'
**Genotyping**		
ANTH3	5'-CACATCTGGGTGGGAGCTTG-3'	5'-GCCATTGGTACGAGGTCACC-3'
GUS	5'-AACGCCTGATCAATTCCACAG-3'	5'-GCCATTGGTACGAGGTCACC-3'

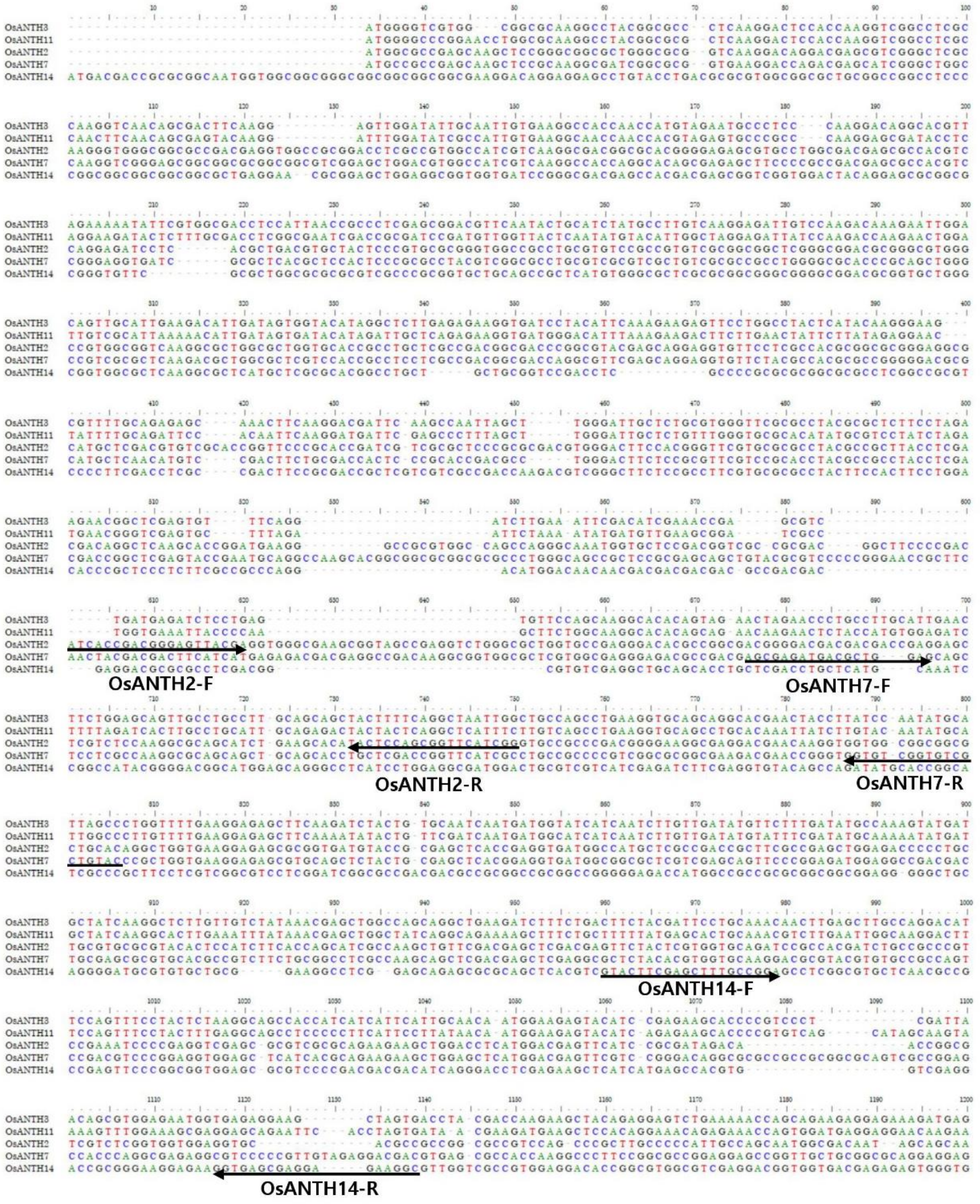
OsANTH3
OsANTH11
OsANTH2
OsANTH7
OsANTH14
OsANTH2-F
OsANTH7-F
OsANTH2-R
OsANTH7-R
OsANTH14-F
OsANTH14-R

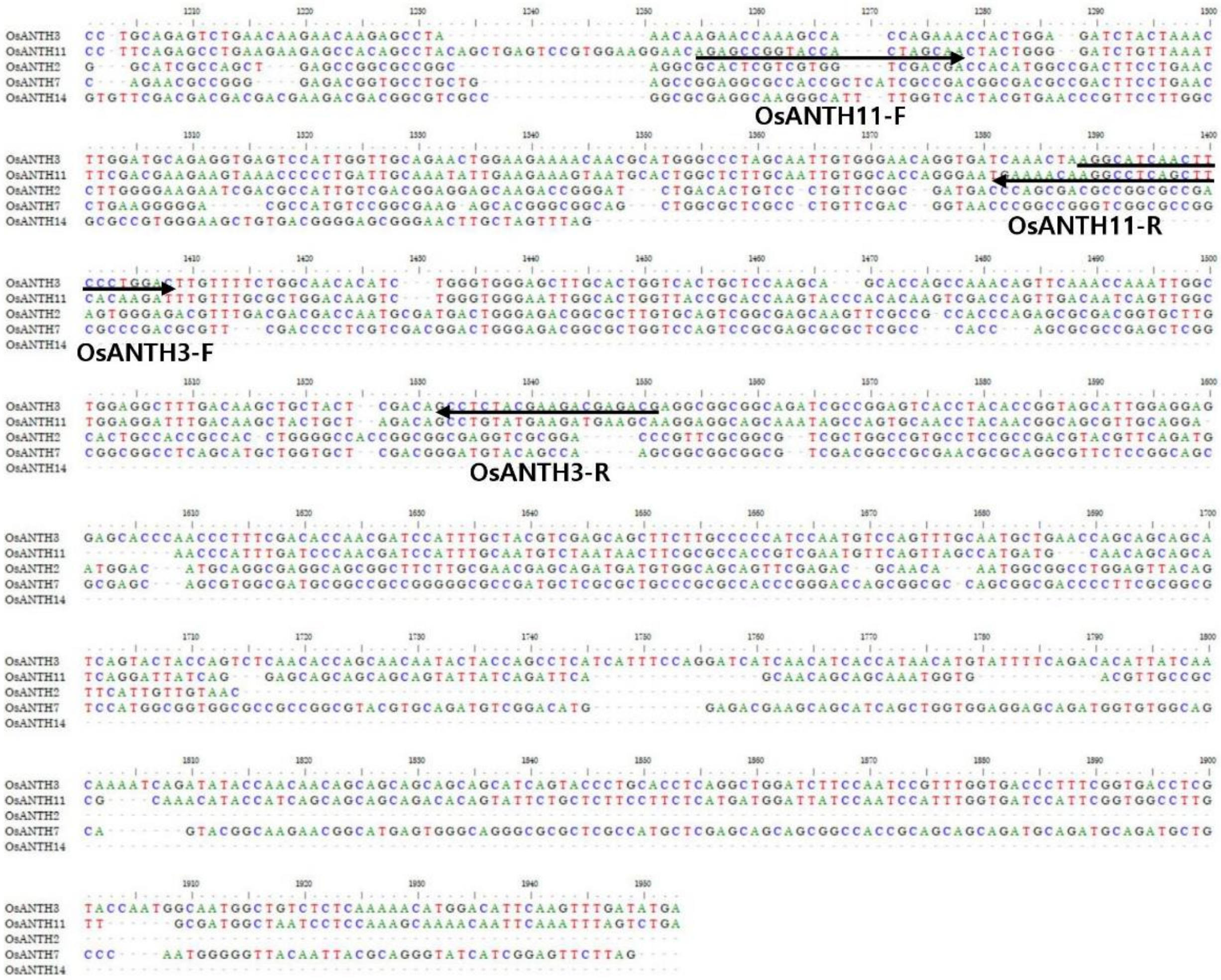

Figure S1. Primer design for qRT-PCR. Nucleotide sequences of pollen-preferred genes encoding ANTH domain-containing protein in *Oryza sativa* (*OsANTH3* and *OsANTH11* genes) were aligned using ClusterW program. Arrows indicate the positions of gene-specific primers used for qPCR analysis.

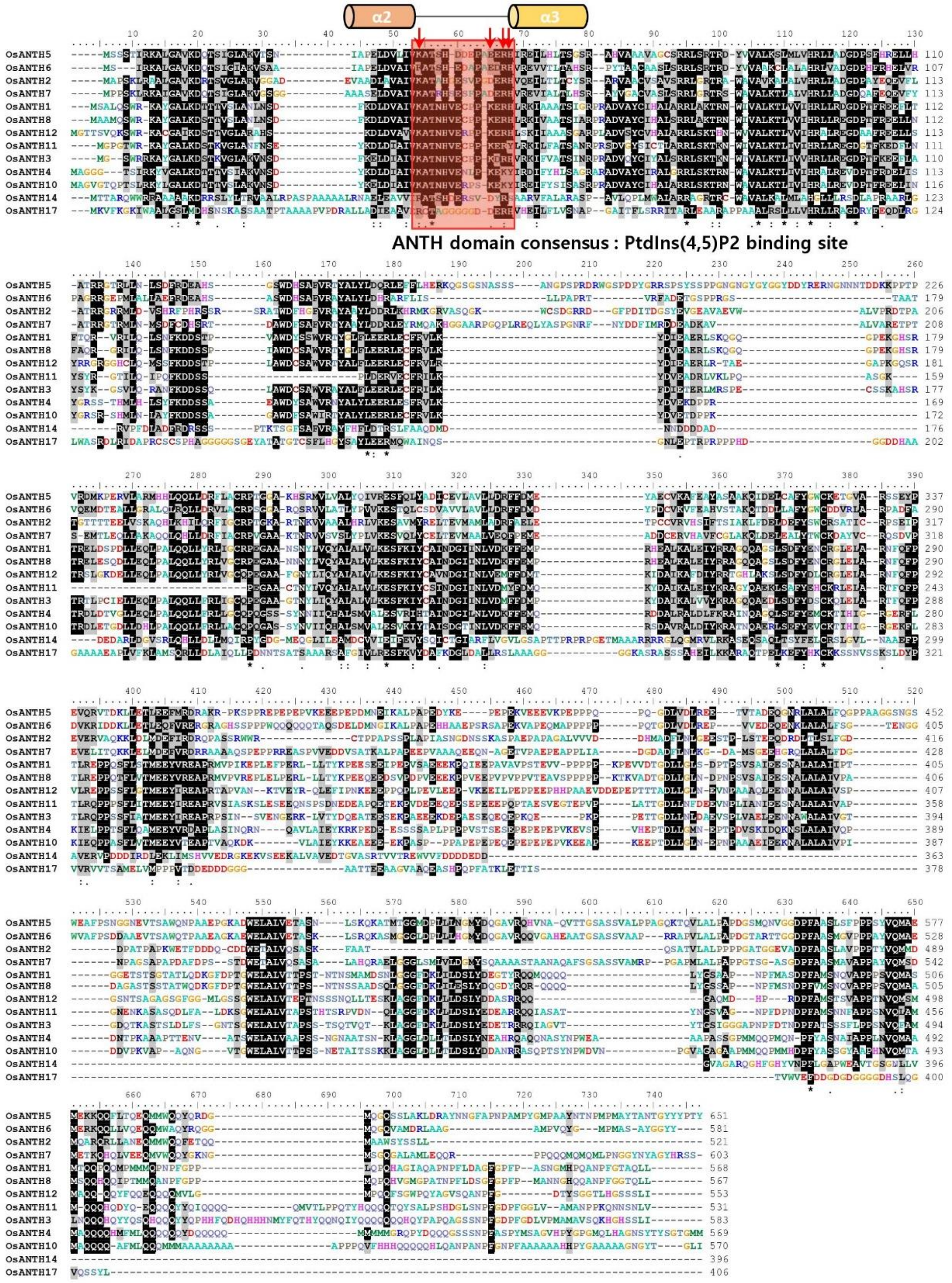

Figure S2. Protein sequence alignment domain analysis and conserved phosphorylated amino acid of protein sequences encoded by Os*ANTH* genes. 13 OsANTH protein sequences were collected and aligned. ANTH signature motif, KAT(X)5/6P(X)3K/RH/Y, is well conserved between α-hellix 2 and α-helix 3 modifs, except partial conservancy in OsANTH14 and OsANTH17. The red arrow indicates the key PtdIns(4,5)P_2 binding residues. The C-terminal part of the amino acid alignment is less preserved than that of the N-terminal part.

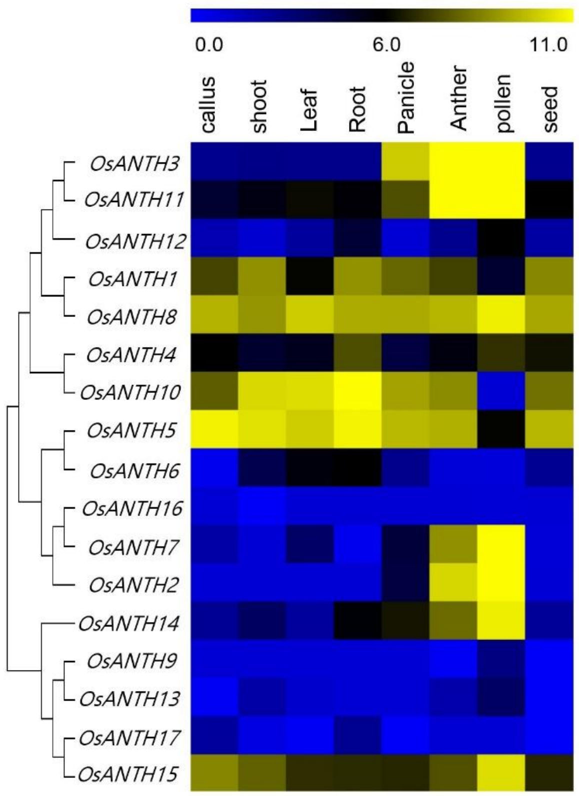

Figure S3. Heatmap expression analysis of all 17 *OsANTH* genes in eight tissues/organs. Phylogenetic tree of *OsANTH* genes were combined with heatmap of RNAseq data. Yellow color in heatmap indicates a high level of expression; dark blue, low expression. Numeric values indicate an average of the normalized $\log_2$ intensity value of microarray data.

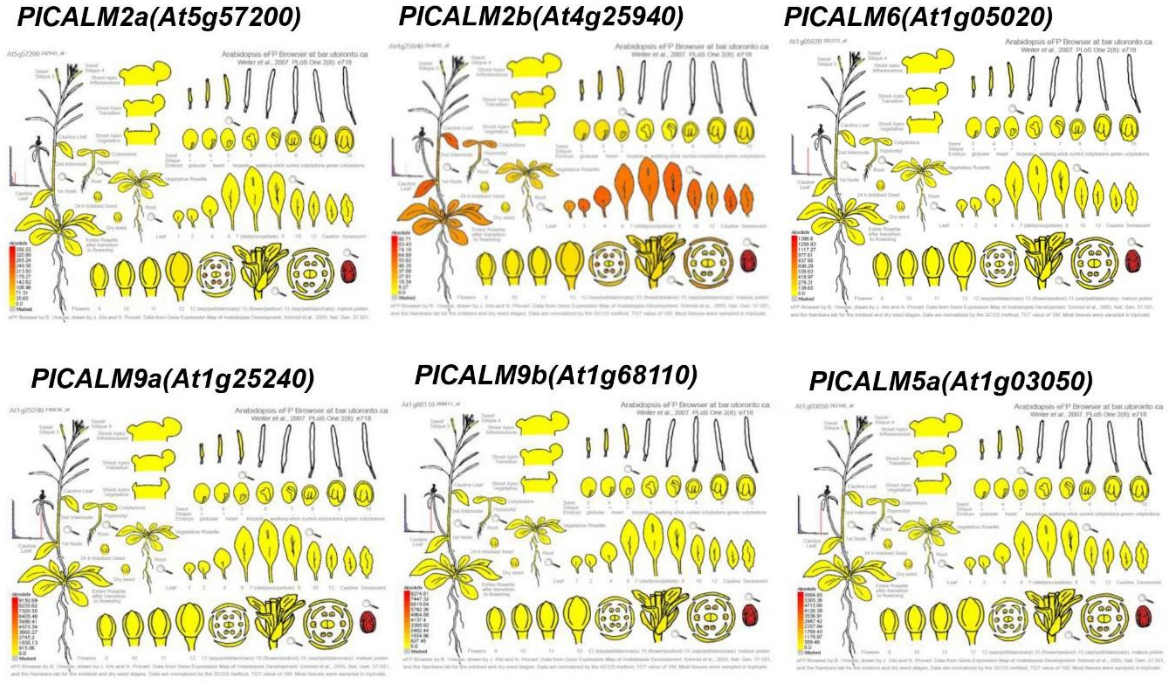

Figure S4. Pollen specific expression patterns of ANTH gene (PICALM2a, PICALM2b, PICALM6, PICALM9a, PICALM9b, PICALM5a) in Arabidopsis thaliana from the public database (http://bar.utoronto.ca/efp/cgi-bin/efpWeb.cgi). Data from gene expression map of Arabidopsis development based on microarray and RNA-seq. These expression pattern suggests the role of the *ANTH* genes in the rice, which has a close relationship with the above pollen preferred Arabidopsis genes.

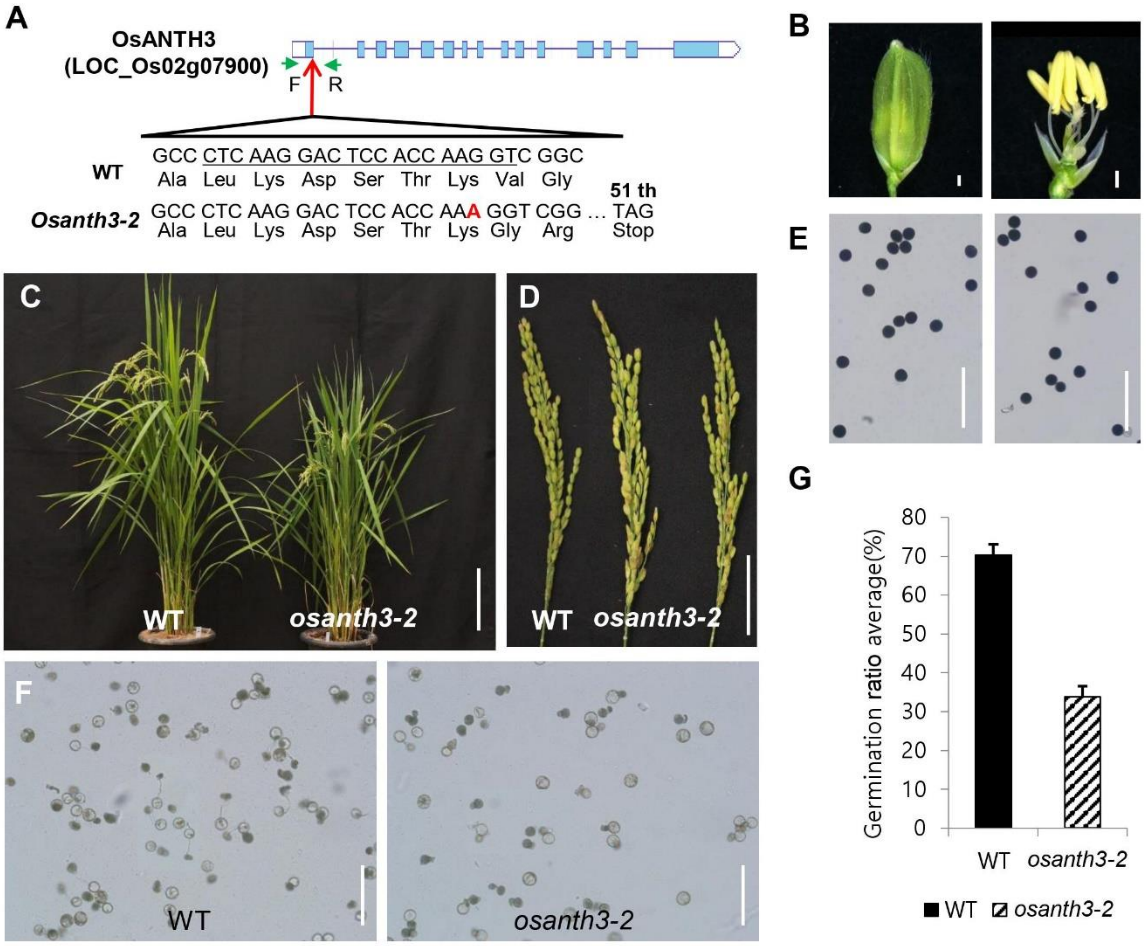

Figure S5. Phenotypes by the mutation of *OsANTH3* (*LOC_Os02g07900*). Pollen-preferentially expressed *OsANTH3* gene was mutated by using CRISPR/Cas system (A) Schematic diagram of *OsANTH3* in *osanth3-2* mutant. Adenine is inserted in the first exon of the *OsANTH3* gene. (B-E) The appearance of the whole plant, panicle, spikelet and pollen between wild type (left) and *osanth3-2* mutant (right). Pollens of wild type (left) and *osanth3-2* mutant (right) were stained with KI solution (F). (G) The germination rate was calculated as (the average of the number of pollens normally germinated) /(the number of total pollen grains)x100, n=100. Scale bars, 10 µm in (B); 10 cm in (C); 5 cm in (D); Bars = 200 µm in (E and F). Error bars represent the standard errors of three biological replicates (G).

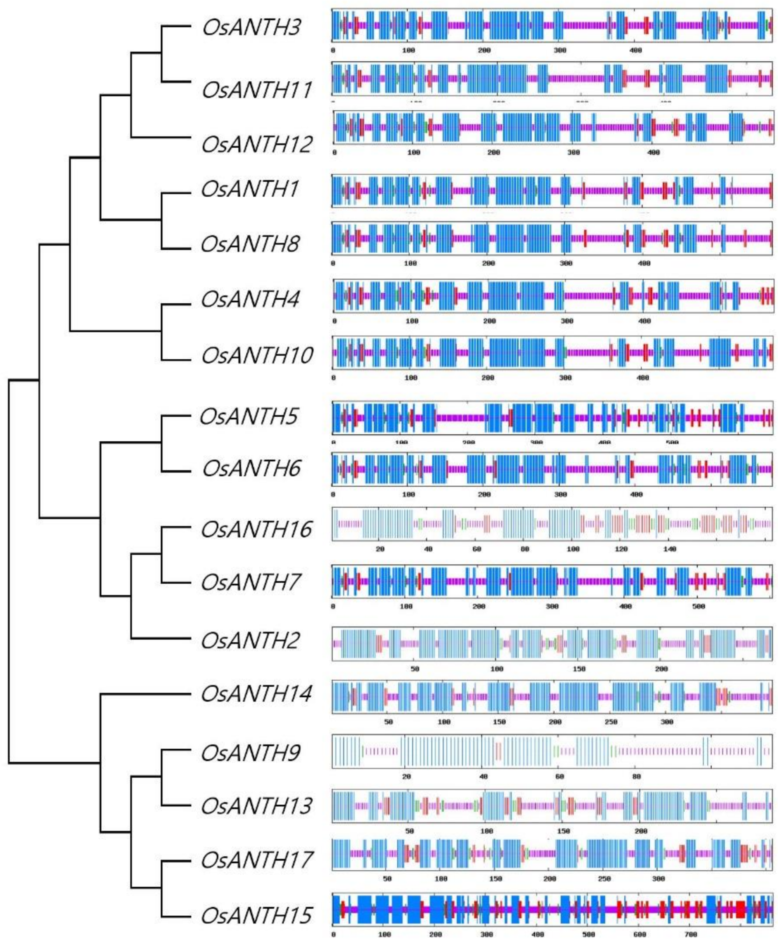

Figure S6. Secondary structure of OsANTH proteins using the deduced amino acid sequences. Blue lines indicate alpha helices, purple lines indicate random coils, red lines denote extended strands, and green lines represent beta turns.

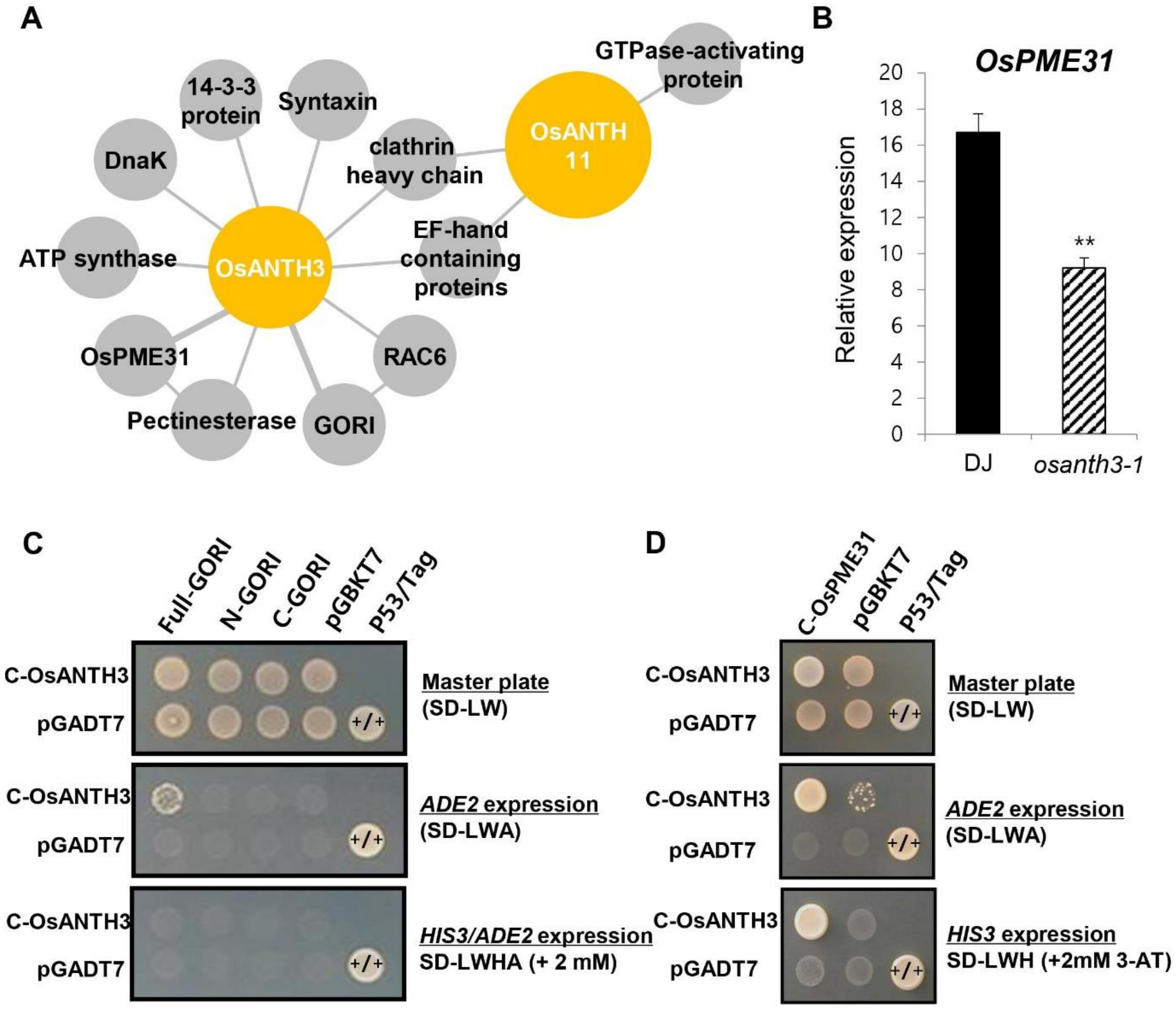

Figure S7. (A) Gene network associated with OsANTH3 using string database (https://string-db.org/) and bar database (http://bar.utoronto.ca/). (B) qRT-PCR expression analysis for *PME31* in DJ and *osanth3-1*, *P<0.01. Yeast two-hybrid assay to test the strength of the(C) interaction of C-OsANTH3 and GORI and (D) C-OsANTH3 and OsPME31. C-OsANTH3, C-terminal region of OsANTH3 from 1096aa to end). *AH109* yeast transformants were dropped onto selective medium lacking Leu and Trp (SD-LW) or also lacking Ade (SD-LWA) or Ade and His (SD-LWAH), with or without 5 mM 3-amino-1,2,3-triazole (3-AT), a competitive inhibitor of yeast HIS3. Positive control, yeast transformed with the P53 bait plasmid and the Tag prey plasmid. Negative control, yeast transformed with the parental bait vector (pGBKT7) and the prey vector (pGADT7).
